# Meat Analogues in the Perspective of Recent Scientific Research: A Review

**DOI:** 10.3390/foods11010105

**Published:** 2021-12-31

**Authors:** Klaudia Kołodziejczak, Anna Onopiuk, Arkadiusz Szpicer, Andrzej Poltorak

**Affiliations:** Department of Technique and Food Development, Institute of Human Nutrition Sciences, Warsaw University of Life Sciences, Nowoursynowska 159c Street, 32, 02-776 Warsaw, Poland; kolodziejczakklaudia@gmail.com (K.K.); arkadiusz_szpicer@sggw.edu.pl (A.S.); andrzej_poltorak@sggw.edu.pl (A.P.)

**Keywords:** meat alternatives, plant-based meat, consumption, consumer acceptance

## Abstract

There are many reasons why consumers and food producers are looking for alternatives to meat and meat products, which includes the following: health, environmental or ethical aspects. This study reviews recent scientific reports on meat analogues. The scope of the review includes the following: formulation and nutritional value; health safety and legal regulations; manufacturing and processing technologies including the latest developments in this area; product availability on the food market; and consumer attitudes towards meat analogues. The analysis of the literature data identified technological challenges, particularly in improving consumer acceptability of meat analogues. Among the risks and limitations associated with the production of meat analogues, the following were identified: contamination from raw materials and the risk of harmful by-products due to intensive processing; legal issues of product nomenclature; and consumer attitudes towards substituting meat with plant-based alternatives. The need for further research in this area, particularly on the nutritional value and food safety of meat analogues, was demonstrated.

## 1. Introduction

The world is constantly changing. These relate to the environment human population structure, the state of natural resources and biodiversity. Importantly, consumer awareness is also changing, resulting in a shift in dietary choices. Many different factors have been identified as contributing to the need to reduce meat consumption, both individually and globally. However, despite growing consumer environmental and health awareness [1,2,3], the proportion of the population that chooses switch to a meat-free diet is a minority (approximately 5% vegetarian diet and 3% vegan diet) [3]. This percentage is growing intensely, as is the number of people on a flexitarian diet limiting their dietary meat intake [2,3,4]. One strategy to support consumers in their decision to reduce meat consumption is to offer them products that replace meat. Among these products are meat analogues.

One of the most important reasons for limiting meat consumption is the growing human population. In 2017, the number of people in the world was 7.5 billion, but estimates by the United Nations [5] show the population growing to 8.5 billion in 2030 and even 10 billion in 2050. Currently, population growth has resulted in intensified food production, and this has resulted in unsustainability in the current food system. The observed rapid population growth coupled with the inefficiency of current dietary patterns generates an extremely important problem, namely, feeding such a large number of people [6]. Malnutrition is largely caused by a protein deficit rather than insufficient calorie intake. Conversion of plant protein to animal protein is characterized by low efficiency and high losses of protein and energy [6,7]. For example, 6 kg of plant protein is needed to produce 1 kg of meat protein [8]. Animal production is, thus, insufficiently efficient, and it becomes crucial for food security to look for alternative protein sources: plant protein, protein from insects or protein from unicellular organisms [9,10]. No less important motivation for change is the environmental impact of animal production. Among other things, the intensive livestock production system results in a significant loss of biodiversity, which is caused by the huge demand for land for feed crops [11,12]. Ammonia emissions; progressive deforestation; and disruption of phosphorus, nitrogen and carbon cycles also have a negative impact on the environment [12]. Although the alarming state of the environment and availability of key resources (i.e., drinking water), as well as food security, undoubtedly justifies the need to reduce meat consumption, many consumers are unaware of the scale of these problems.

Health concerns are also among the arguments for reducing meat consumption. There is a known link between the consumption of processed meat products and an increased likelihood of certain diet-related diseases: obesity, type 2 diabetes, cardiovascular diseases, colorectal cancer or strokes [13,14,15]. We should also not forget the risks of zoonotic diseases, i.e., avian influenza and human CJD (Creutzfeldt-Jakob Disease) associated with bovine BSE (Bovine Spongiform Encephalopathy or mad cow disease), Q fever, SARS (Severe Acute Respiratory Syndrome) and MERS (Middle East Respiratory Syndrome) [7,9,13]. Another health risk associated with livestock production is the increasing antibiotic resistance of pathogenic microorganisms (including methicillin-resistant *Staphylococcus aureus*) [7]. This is largely due to the massive use of antibiotics in animal husbandry [2]. It is estimated that there will be a further intensive increase in antimicrobial use, with 2/3 of this increase occurring in the livestock sector [16]. This is of great concern as it could result in the emergence of highly dangerous strains of antibiotic-resistant bacteria threatening the health of the population [17,18]. A large proportion of consumers choose to exclude meat from their diet for ethical reasons and due to concerns about the welfare of farmed animals [1,19]. Some consumers are driven to switch to a plant-based diet by fashion or the desire to adapt to their environment.

The total or partial abandonment of meat in the diet, regardless of the reason, requires the supply of an adequate amount of protein from other sources. So-called meat analogues can be particularly helpful, especially at the initial stage of dietary changes. These products aim to replace meat and meat products in their functionality while being similar in terms of sensory properties, particularly taste, aroma and texture and nutritional value [8,20,21]. The growing interest in meat analogues is not only observed among consumers and food producers but also in the scientific community. In recent years, many new scientific studies have emerged on topics such as the following: the formulation and technology of meat analogues, the market situation and consumer attitudes towards plant-based diets and meat replacement products. This study analyses these reports and identifies challenges, risks and limitations associated with the production of meat analogues.

## 2. The Essence of Meat Analogues

### 2.1. Composition of Meat Analogues

The most important nutrient in meat is highly digestible protein containing all essential amino acids. Therefore, the essence of meat analogues is a well-chosen source of protein. Almost all plant proteins can serve as raw material for meat analogues, but there are several important issues that determine their suitability for this purpose. The most important issues to consider are the availability and cost of the raw material and its technological properties. The most widely used building proteins for meat analogues include soybean, pea and wheat proteins [13,22]. These are relatively cheap and available raw materials, but in the case of soy and pea proteins, a significant challenge is to achieve the fibrous structure that is a characteristic of meat. The key in this case is the fact that they consist mainly of globular proteins, which are not conducive to forming the desired structure [22]. For this reason, plant proteins often require intensive processing and the use of additives to provide a satisfactory product structure. A second important characteristic of plant proteins that affects the composition of meat analogues is the profile of essential amino acids. In contrast to meat, plant proteins contain a so-called limiting amino acid (e.g., lysine in cereal proteins) [23]. In order to achieve a balanced amino acid profile in the analogue recipe, the addition of suitable proteins, e.g., rice or mung bean, is used. Moreover, sources of building proteins for meat analogues other than plants should not be overlooked. Over the years, the possibility of using proteins from fungal fermentation (mycoproteins), insects, microalgae and even non-pathogenic bacterial strains, among others, has been investigated [9].

Fat plays an important role in the nutritional value and sensory properties of meat and its analogues. It is responsible for the texture and mouthfeel and is also a carrier of flavour and fat-soluble vitamins. In meat analogues, solid fats extracted from coconut and cocoa and vegetable oils, most commonly rapeseed and sunflower, are used [23,24]. In order to improve the fatty acid profile and taste of the product, oils of different origins are used, i.e., sesame oil and avocado oil [25,26]. New technological developments even make it possible to mimic in burger and sausage analogues the characteristic marbled appearance, e.g., by whipping a mixture of oils into small globules of fat [22,27]. The disadvantage of vegetable fats is that they lack the meat-specific volatile substances embedded in animal fat [22,28]. However, from a nutritional point of view, vegetable fats are more beneficial, mainly due to their fatty acid profile (higher content of unsaturated fatty acids) and lack of cholesterol [13,22].

Meat and meat products are very complex structures that give them their characteristic texture, organoleptic values and nutritional value. In order to impart similar properties to meat analogues, it is necessary to use many different functional components in the formulation of these products. For example, the function of myofibrillar proteins is to build texture and to immobilise water in meat [22]. Their function in meat analogues is performed by carbohydrate polymers. These include ingredients belonging to three groups: plant fibres, starches and polysaccharides and their derivatives. These ingredients are responsible for improving texture, binding water in the product and reducing syneresis. The most commonly used are pectins and polysaccharide gums of various origins, e.g., xanthan [24,25].

When making purchase choices, colour is an important characteristic of the product that determines purchase intention. Soy or gluten proteins are naturally yellow or beige in colour, making them unattractive to consumers [17,24]. The characteristic colour of red meat in analogues is achieved by using extracts from beets containing betaine; carrots and peppers containing beta-carotenes; tomatoes containing lycopene; or berries containing anthocyanins [24,29]. However, the pigments they contain during thermal processing may become discoloured. An ingredient that allows for the brown colour of cooked meat is apple extract [22]. Its polyphenols and ascorbic acid are oxidised during processing, resulting in a change in the appearance of the product [22]. Innovative dyes of biotechnological origin are also used. One example is leghaemoglobin, for which its properties are similar to haeme present in meat. It gives meat analogues a so-called “bloody” appearance [29,30].

Colour is a feature that is very important, but it is the taste that determines the success of the product. In order to replicate the aroma of meat products, a variety of herbs and spices are added to meat analogues, which include black pepper, oregano, sage, paprika, rosemary, cloves and many others [22,24]. Yeast extract, nucleotides and reducing sugars are also used to intensify and improve flavour [24,26]. Much higher amounts of flavour additives are used for meat analogues than in meat products, as they are intended not only to mimic the complex aroma of the products they replace but also to mask the undesirable aftertaste of certain raw materials (e.g., beans) [31]. Various techniques are also used to remove the taste of legumes [32].

From the point of view of the nutritional value of meat, vitamins and minerals are important ingredients in the formulation of a meat analogue. Particularly important are vitamin B12 and iron and zinc, and deficiencies may occur in plant-based diets. For this reason, plant-based alternatives are fortified to achieve a quantity and proportion of nutrients similar to meat [4]. The range of additive ingredients used in meat analogues is very diverse and growing. Examples include enzymes such as the following: transglutaminase, which ensures strong protein binding in textured plant protein products [24,33]. Plant analogues and, thus, the proteins and fats they contain undergo intensive processing. For this reason, antioxidants are added for which its function is to prevent rancidity of the fat and oxidation of the protein. Substances such as sodium nitrite used, e.g., in conventional meat products, are a source of controversy because of their influence on human organism. Therefore, alternatives are being studied for use in meat and meat analogues. Plant extracts are an alternative to synthetic antioxidants that can be used by food manufacturers. Antioxidant compounds of plant origin are chemically heterogeneous group. The most widely used in the meat industry are polyphenols (flavonols and anthocyanins) and essential oils (mainly terpenoids), which are used as a product ingredient or packaging element [34,35]. Bioactive compounds are extracted from different parts of plants (leaves and seeds) such as pepper (*Piper nigrum* L.), oregano (*Origanum vulgare* L.) or juniper (*Juniperus communis* L.) [34,35]. There are many studies on the use of natural antioxidants to extend the shelf life of meat products. Thus, there is a need for research on the use of these compounds and also in meat analogues [34]. Organic acids, phosphate compounds and plant extracts are used to ensure the microbiological stability of meat analogues [22].

A product analogous to conventional meat and meat products can also be cultured meat produced using “in vitro” technology. This is a slightly different example of a meat analogue in that it consists of replicated animal cells that have been painlessly taken by biopsy from a living animal. Stem cells are collected and then differentiated into desired tissues under strictly controlled conditions and in the presence of an appropriate medium. Animal serum-based medium is most commonly used to produce cultured meat, but research is underway to develop serum-free medium for safety and ethical reasons [2,9]. Recent research in meat analogues along with their formulations are shown in Table 1.

### 2.2. Technologies for Producing Meat Analogues

Despite many technological advances in the production of meat analogues, the most widely used texturing method for amorphous plant materials to date is extrusion [8,9,10]. The principle of the method is to subject a food mixture containing proteins to hydration and high temperature, high pressure and mechanical interactions [8,41]. The extrusion process consists of three main steps: mixing proteins with water in a twin-screw extruder; cooking in a chamber under high temperature and pressure; and cooling in a cooling matrix [9,13]. Many parameters affect the extrusion process and the product texture obtained, including the following: water content, screw speed, processing temperature, matrix geometry, presence of polysaccharides and type of raw material [13,41]. There are two types of extrusion that result in products with different properties. Low-moisture extrusion (water content up to about 30%) is primarily used to produce textured vegetable protein (TVP). The product of such processing is characterised by a porous, spongy structure, high water binding capacity and low water and fat content [9,26]. TVP produced using low-moisture extrusion requires hydration prior to further processing, e.g., to produce a meat analogue in the form of chunks, nuggets or crumbles [41]. The second type of extrusion is high-moisture extrusion (above about 40%) [9,26,42]. This method is used to produce meat analogues with a fibrous structure resembling whole muscle or restructured meat products [41,42]. High-moisture extrusion allows for more complex formulations, and it is not necessary to use ingredients with high solubility, making it a more cost-effective technology [26,42]. Whether a material can be extruded is primarily determined by the ratio of soluble to insoluble components, which affects the crosslinking process of proteins [8]. Soy proteins, wheat gluten and pea proteins are most commonly extruded, but the extrusion of other plant materials such as cottonseed, rapeseed, peanuts and sesame seeds has also been investigated. It is, therefore, possible to further extend the usefulness of extrusion to produce meat analogues [13].

Plant protein texturing techniques also include wetspinning and electrospinning. These techniques are used to create fibres from protein solutions. In the case of wetspinning, the protein solution is pressed through a spinneret and then dipped into a protein non-solvent. The extruded protein phase is precipitated and solidified. Wetspinning results in fibres of approximately 20 μm thickness [8,43]. Due to the use of many chemical reagents, this technique generates large amounts of waste, which in turn limits its use [26]. In terms of sustainability of food production, electrospinning is a more attractive technique. This method involves pumping a protein solution through a hollow needle or spinneret at an electrical potential relative to a grounding electrode. An electrical charge accumulates on the surface of the droplets, which results in instability of their surface. The result is the transformation of the protein solution into very thin fibres that are attracted to the ground electrode. The electrospinning technique has been used in the production of nanofibres but is a promising tool in the production of meat analogues from whole muscle [8,43]. Three-dimensional printing, otherwise known as fused deposition modelling (FDM), is also an innovative technique for producing meat analogues [44]. This is a method of so-called additive manufacturing, for which its potential applications include creating meat-like structures from whole muscle. The basic components of a food 3D printer include the following: a platform attached to a stage and a head (most commonly an extruder type) driven by a motor [44,45]. There are different 3D printing techniques: extrusion, inkjet printing, binder jetting and bioprinting. The production of meat analogues most often uses extrusion-based 3D printing, which involves extruding a material, consisting of a mixture of, e.g., plant protein and water through a nozzle and forming the product layer by layer in a manner that mimics muscle fibres [22,41]. The process is influenced by the printability of the material used, which ensures the flow out of the nozzle and the ability to maintain and stiffen the 3D structure after extrusion [41,45]. Various components (e.g., transglutaminase) are added to the extruded material, for which its function is to produce the desired firmness and improve its rheological properties. Among others, soybeans, wheat, peas, mushrooms and insects are used as materials for 3D printed meat analogues [44]. The use of extruder-type 3D printers to produce meat products with modified properties, e.g., adapted for the needs of the elderly has also been investigated. This provides an opportunity to extend the use of 3D printing technology to the production of cultured meat analogues [45]. However, for in vitro produced meat, bioprinting is a more promising 3D printing method [44]. Three-dimensional printing is a novel technique that requires further research with a view with respect to sustainable meat analogue production and process optimisation [9]. Among the techniques for the production and texturing of meat analogues, the shear cell technique is also noteworthy. It is based on the concept of flow-induced structuring in which intensive shearing of the plant material takes place. The texturing process takes place in cone-in-cone or Couette cell devices, with the use of a Couette cell being particularly promising in terms of yield and the possibility of increasing the scale of production [42,43].

## 3. Market Development for Meat Analogues

Consumption of plant-based protein products has a documented history dating back to antiquity. The oldest known alternatives to meat are tofu and tempeh [17,43]. These are soy products originating from Asian countries that became widely available in Western countries in the 1960s [10,31]. Traditional tofu production involved coagulating soy “milk” with salt or acid. The curd thus formed was pressed into blocks with a light colour and a characteristic aroma. Over the years, the production method has been improved and different flavour and texture variants of this product have appeared on the market [17,46]. Tempeh, which originates from Indonesia, is also produced from soybeans. Tempeh is made from soaked, partially cooked soybeans, which are then fermented by *Rhizopus* fungi. During fermentation, oxygen is restricted, and the developing fungi cause beans to solidify into a uniform block. In addition to soy products, seitan (wheat gluten) has a long history of use as a meat analogue. It is a product made from wheat flour by leaching starch out of it until an elastic mass consisting only of gluten is obtained. Seitan, similarly to tofu, originates from Asia. Its main advantages are its structural similarity to meat and its ability to be shaped to resemble a given meat product (e.g., chicken wings) [17]. 

All three products described are readily used by vegans and vegetarians in meals as meat analogues, but they differ from meat in terms of texture, taste and aroma. The low sensory appeal of these products, understood as a low similarity to meat, is the main reason for their low popularity among flexitarians and omnivores [31]. The increased interest in plant-based diets and the growing demand for protein products as an alternative to meat contributed to the development of textured plant protein in the second half of the 20th century. Textured vegetable protein is obtained by extruding defatted soybean meal, soy protein concentrates or wheat gluten [43,46]. The name textured vegetable protein originally protected by a trademark has over time become a term for an entire product category. They are used as an ingredient in meatless versions of dishes in the role of burger, bacon or minced meat [17]. Textured vegetable protein gave rise to the concept of meat analogues, which was intensively developed in the following years.

Similarly to tofu, tempeh and seitan, textured vegetable proteins are usually accepted by people who exclude meat from their diet (vegans or vegetarians). Their sensory properties may not be satisfactory, particularly for carnivores and flexitarians who value the properties of meat and expect them to be reproduced as closely as possible by plant-based alternatives [9,17,31]. With technological development and increasing consumer demands, a dynamic development of the category of products alternative to meat is observed. The latest generation of meat analogues is characterised by very similar aroma and texture to the products they are supposed to replace [47]. Innovative solutions are being used, making it increasingly difficult to distinguish a plant-based product from a conventional one [10,46].

The current range of meat analogues is very wide. There is great diversity in terms of product types but also in terms of recipes and characteristics. The media have declared 2019 the year of the plant-based burger, which is the most recognisable and constantly improving analogue product. However, analogues of sausages, frankfurters, cold cuts, pates, bacon, chicken wings, various types of meat chops and cuts and even seafood are also available for sale [17,41,46]. Although these products constitute a distinct category, it should also not be forgotten that in recent years a technique to produce “meat” by using in vitro cultivation has been developed, which in the future could be a promising option for welfare-sensitive consumers of slaughter animals who do not want to exclude meat from their diet [9,47].

**Figure 1:**
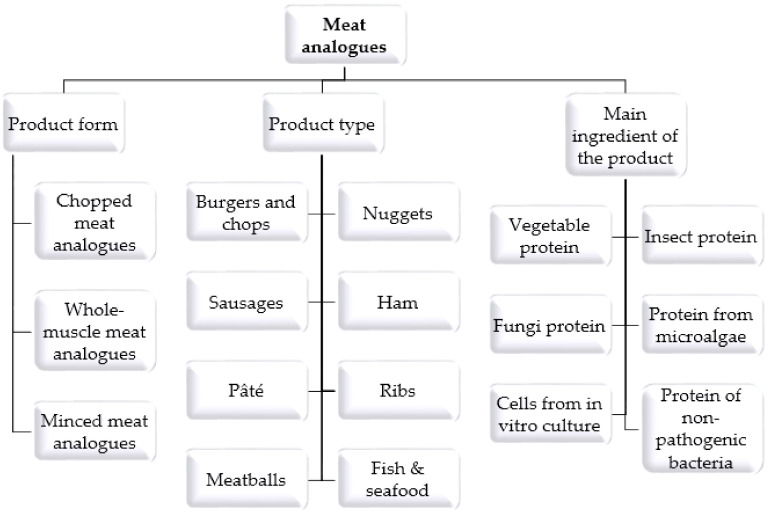
Types of meat analogues available on the market.

It is estimated that the number of products in the meat analogue category has increased fivefold in just four years and includes more than 4400 different products [4]. The best-selling meat analogue categories are burgers, sausages and patties [6]. Examples of commercially available meat analogues are shown in Figure 1. The meat analogues market is projected to grow at an expected annual growth rate of 7.9% from 2019 to 2024, making it estimated to be worth USD 21.23 billion in 2025 [10,25,48]. Compared to projections for the meat industry, which could be worth up to $7.3 trillion by then, these figures are small. Meat analogues, thus, represent a promising and rapidly growing, but it is still niche food category [25,49,50]. It is interesting to note that the largest number of companies producing meat analogues are based in North America, slightly fewer are located in Europe and only a few are located in Asia, Australia or Africa. However, data show that the fastest growing market for meat analogues is precisely the Asian region, while Europe is the largest [25]. Most of these companies started within the last 10 years [6].

Some of the best-known examples of brands that have been successful in the analogue market include the following: Beyond Meat™, Impossible Food™, Light Life™ and Gardein™ [17,22,46]. A big influence on the dynamics of these companies and the overall meat analogue market is the funding of plant-based start-ups by investors such as Bill Gates, huge companies (e.g., Cargill) and various incubators. Moreover, these companies have both B2B (business-to-business) and B2C (business-to-consumer) activities. The B2B model is particularly influential in the popularisation of meat analogues, as it makes it possible to attract consumer attention to the products offered. An example of the use of the B2B model is the launch of the Impossible Whoppers burger developed in partnership between Burger King and Impossible Foods. The success of this burger resulted in the expansion of the restaurant menu to include Impossible Pork and Impossible Sausage. Moreover, the Beyond Meat brand was successful in the B2B model when the KFC chain offered a plant-based chicken wing analogue developed by them to customers [6,22,43]. Companies previously involved in meat production have also had their share of the meat analogue market and have expanded their product range to include plant-based analogue products. The interest of food manufacturers in meat analogues is also indicated by the fact that many grocery shop chains (e.g., Kroger) are introducing private label plant-based meat analogues [22].

## 4. Consumer Attitudes towards Meat Analogues

It is extremely difficult to describe consumer preferences or attitudes towards meat analogues. The limitation of the majority of conducted scientific research is, above all, the possibility to examine only small sections of the population, most often narrowed down to a specific region or social group. It should, therefore, be taken into account that inference involves generalisation, which may be subject to certain error. This research is, however, very important for understanding consumer preferences and motivations in making purchase decisions. The results obtained influence the possibility of developing appropriate marketing or information strategies, which aim, among others, to encourage consumers to reduce meat consumption, which is desirable for the reasons cited in the introduction to this review. This section presents the results of studies published in the last 3 years, as older studies are included in other reviews, e.g., Hartmann and Siegrist [51] and He et al. [17]. A summary of recent scientific research is presented in Table 2.

At the outset of considering consumer attitudes towards meat analogues, it is worth looking at who the consumers on vegetarian, vegan and flexitarian diets, who consume meat analogues, actually are. It is worth citing at this point the results of a study conducted by Milfont et al. [52] on a large sample of New Zealanders. The aim of the study was to identify predictors of eating behaviour. Results showed that meat eaters were the largest group (94.1%). It was observed that higher levels of conservative ideology and lower subjective health status were associated with adherence to an omnivorous diet. The analyses also showed that political conservatism and gender (male) correlated with a low likelihood of switching from a meat to a meat-free diet. Thus, those following a meat-free diet were more likely to be female, have more liberal political views, be prone to disgust, display a pro-social attitude and be open to new experiences. Meat loathing was examined by Becker and Lawrence [19]. This study measured declared meat consumption, level of self-control, liking for meat, disgust sensitivity and overt and covert meat loathing. In the study group, 57% of the respondents were omnivores, 28% flexitarians and 15% people on a plant-based diet. The study showed that there was a negative correlation between loathing of meat and meat consumption; moreover, this relationship was most pronounced in flexitarians. Importantly, not all vegetarians were classified as showing meat loathing. The researchers also analysed changes in perceived meat loathing and meat consumption over time. It was shown that developing a stronger disgust for meat correlated with a reduction in meat consumption but only in relation to overt disgust. Again, this relationship was strongest in the flexitarian group. These results indicate not only that people on a plant-based diet and flexitarians are two distinct population groups and should be analysed as such but also that strategies to encourage a reduction in meat intake based on disgust are most likely to be successful in flexitarians. An interesting study was conducted by Davitt et al. [53]. They examined, among other things, the differences between attitudes towards spirituality, vegetarianism, environmental sustainability, environmental awareness and nutrition among eaters and non-eaters of plant-based meat analogues. The results showed that people who did not consume meat analogues were more likely to consider themselves religious. They were also more likely to agree with the statement that dinner without meat is not a proper meal and that vegetarians are “a bit different”. Those who consumed meat analogues were more likely to agree with the statement that these products were less harmful to the environment and that they provided an adequate amount of protein. 

Many studies have focused on determining consumer preferences for different types of products as meat alternatives. In the vast majority of studies, plant-based meat analogues are more acceptable than products based on lesser-known protein sources such as insects or in vitro cultured “meat” [43,49]. Very low acceptance towards insect proteins is largely related to dietary neophobia [54]. A study by Slade [55] showed that when given a choice between equally tasting beef, plant-based and in vitro cultured meat burgers, consumers were most likely to choose the beef burger. Only 21% would buy a plant-based burger and 11% would choose a burger made from “cultured meat”. In a study by Bryant and Sanctorum [2] involving Belgian consumers, it was shown that there were differences between consumers who declared positive attitudes towards plant-based meat analogues and those who would choose in vitro cultured meat. Plant-based analogues were significantly more attractive to women and those on a meat-free diet, while in vitro cultured meat was a more attractive option for men and meat eaters. Over 40% of respondents are positive about alternatives to meat, but this group includes two disconnected subgroups: consumers who prefer plant-based alternatives and those who choose cultured meat. This indicates a legitimate need for a variety of alternatives to meat in the market. The same study found a significant increase in Belgian consumers’ satisfaction in having their expectations met by plant-based meat analogues in 2019 (44%) and 2020 (51%). Higher satisfaction with available meat analogues was positively correlated with belonging to a younger age group, gender (female) and following a meat-free diet.

A positive attitude and enjoyment of meat analogues is not enough. It is worth considering the amount of consumption of these products and the reasons for their consumption. In a study by Davitt et al. [53], as many as 55% of Midwest University students aged 18–30 consumed meat analogues. When asked about the reasons that led them to consume these products, respondents most often indicated the following: liking to try new foods (66.4%), curiosity (54.1%) and encouragement from loved ones (40.3%). Nearly a third of respondents indicated that they try to eat less meat and that plant-based alternatives are better for the environment. Only 20–25% cited health, animal welfare or cost as reasons. This is a surprising result, as the Bryant and Sanctorum [2] study showed a statistically significant increase in concern about animal welfare issues. Health (82%), sustainability (58.4%), animal welfare (54.3%) and the environment (54.1%) were most frequently cited by consumers among the most important factors influencing their purchasing decisions. Consumers’ food choices are extremely difficult to predict due to the very large number of factors influencing them. Studies indicate that attitudes towards meat analogues can be influenced by determinants such as age, gender, education level, origin, product type, situational context, perceived norms, choices of those around the consumer, religious and political beliefs, economic situation, health status, availability of information, lifestyle, traditions followed and many others [2,19,29,49,54,56,57,58]. Given the need to reduce meat consumption argued by environmental issues, sustainability and population health, it is worth considering what factors limit a reduction in meat consumption and an increase in the consumption of meat analogues among consumers. According to the research, the most relevant barriers were scepticism about the quality of meat analogues, a feeling of lack of skills in preparing meat-free meals, positive associations with meat, health concerns and the need to feel in control of food choices. Consumer uncertainty about the environmental impact of animal production was also demonstrated. The information gathered indicates actions that need to be taken to raise awareness and reduce concerns in consumers [20].

**Table 2:** Research on consumer attitudes towards meat analogues, meat and plant-based diet.

Study Subject	Participants:	Main Parameters:	Reference
meat disgust	711 participants from UK	age, gender, disgust sensitivity, self-control, meat intake, hunger level, english level, meat disgust dummy (% meat disgusted), diet	Becker and Lawrence 2021 [19]
plant-based and cultured meat	2019: 1001 and 2020: 1000 participants from Belgium	age, gender, diet, region, education, rural/urban, satisfaction with existing meat analogues, concern for: animal welfare, impact on the environment, sustainability of choices, health, purchase intent for cultured meat, cultured meat meets their needs	Bryant and Sanctorum 2021 [2]
plant-based meat analogues	1434 Midwest University Students (USA)	age, gender, race, residency, fruit and vegetable servings per day, diet, environmental values, beliefs, knowledge, spirituality, views about vegetarianism, factors influencing food purchase, trusted sources of nutrition knowledge, meat analogues consumption	Davitt et al. 2021 [53]
insect-based and plant-based protein	3091 participants from 9 countries	food neophobia, food tech neophobia, healthiness influence, environmental impact, influence, meat nutritional importance, meat taste, texture, smell importance, plant-based and insect-based protein suitability/benefits, plant-based and insect-based protein willingness to try, buy and pay more	De Koning et al. 2020 [54]
plant-based diet	data from the NZAVS ^1^ 2017: 17,072 participants 2018: 47,951 participants from New Zealand	self-reported dietary behaviour, protection of native species, subjective health, perceived environmental efficacy, gender, political conservatism, right-wing ideology, disgust, religious (spiritual), beliefs, pro-social orientation, openness orientation	Milfont et al. 2021 [52]
plant-based and cultured meat burgers	533 participants	frequency of purchase (meat, burgers and meat substitutes), importance od factors in purchase decision, support for food technology, attitudes towards: agriculture, naturalness of food, lab food, environmental impact of meat, food choices, political views, science, emotional decision making, age, gender, education, income, diet	Slade 2018 [55]

^1^ New Zealand Attitudes and Values Study.

## 5. Challenges and Perspectives

Meat analogues are a product of both hope and concern. It represents an opportunity but also a major challenge for food entrepreneurs. The first major issue regarding meat analogues that is controversial is their labelling. The expanding range of meat alternatives has drawn the attention of consumers, businesses and regulators to the issue of naming meat analogues. Some stakeholders have requested a ban on labelling meat analogues as “meat” and on the use of terms associated with meat, i.e., “burger” or “ham”. The argumentation for such a ban mainly included the risk of misleading consumers [4]. In 2018, in the US, the first state—Missouri—banned the use of meat-related terms for plant or insect protein products. In the following years, this ban took effect in another 25 US states [29,47]. In 2017, the terms “yoghurt”, “cheese” and similar terms were banned in the European Union for products not made from milk. Two years later, a discussion started regarding the labelling of meat-free products with meat-related words. However, in 2020, there was a vote in the European Parliament which decided that producers of plant-based alternatives to meat could use meat-related terms in the marketing and labelling of their products. This indicates a slightly more liberal approach by policy makers in the EU regarding alternative protein sources [2]. This is in line with the “EU Protein Plan” introduced in 2018, which aims to encourage the production and exploration of plant-based alternatives to animal protein [29].

The marketing of new products is also regulated by legislation. In the case of meat analogues based on plant proteins, there are usually no difficulties in placing the product on the market. The ingredients in these products are often pre-authorised for human consumption and widely used. The procedure changes when ingredients not previously used in food production are included in the recipe of the analogue. In the US, the introduction of new ingredients to the market requires approval and designation as “Generally Recognised as Safe (GRAS)”. An example of such an ingredient is soy leghaemoglobin, for which its production process involves genetic engineering. This ingredient had to be assessed and recognised as GRAS by the FDA (Food and Drug Administration) before being marketed in plant-based burgers [6,9,29]. In the EU, such innovative ingredients are subject to novel food legislation [59]. These regulations also cover in vitro cultured meat, insect protein and protein from single-cell organisms. In 2021, the EFSA (European Food Safety Authority) Panel on Nutrition, Novel Foods and Food Allergens issued a positive opinion on the safety of *T. molitor* for human consumption [9].

According to the principles of Regulation 2015/2283, new foods approved for human consumption must be safe for consumers; appropriately labelled; and not be different from the food they are intended to replace in such a manner that their consumption would be nutritionally disadvantageous for the consumer [59]. These principles highlight two further extremely important issues for meat analogues: nutritional value and safety. Meat analogues are seen as a healthier alternative to meat, and labelling with terms associated with meat further suggests that their nutritional value is similar to it. However, opinions in this regard are highly divided [4,6]. The essential amino acid profile of plant proteins seems to be the most relevant. Meat contains all nine essential amino acids, whereas only soy and quinoa among plant ingredients contain all of them but in lower amounts. For this reason, it is necessary to optimise the amino acid content of recipes, e.g., by mixing different plant proteins in the right proportions. An additional issue of concern is the digestibility of plant proteins, which is significantly lower than animal proteins. The exception is soy proteins [6]. In order to ensure similar nutritional value of analogues to meat, attention should also be paid to the content of ingredients of which meat is an important source. Vitamin B12, zinc and iron appear to be particularly important [4]. There are many fortified products available on the market, but there are also products that have not been enriched in these ingredients. A high intake of products that are not enriched, for example, in vitamin B12 in a plant-based diet, can result in deficiencies of this vitamin. One should also not forget the differences in bioavailability of vitamins and minerals. An example of this is non-haem iron, the bioavailability of which is low compared to haem iron. All these factors should be taken into account by meat analogue manufacturers when developing new products [22,30,60].

There is no doubt that the taste and texture of modern meat analogues has improved considerably compared to the first of such products. Further development of meat analogues in this respect is anticipated. The structure and flavour of meat are very complex, which poses a great technological challenge. Food manufacturers use many modern texturing techniques and functional ingredients that impart meat-like sensory properties to analogues to enhance consumer satisfaction [6,22]. However, the focus on creating the closest possible imitation of meat has taken its toll on other aspects of meat analogue quality. The often very long list of additives in product formulation can be a cause for concern [50]. The number of ingredients and additives, as well as salt content, varies depending on the product analysed. Significant differences are also observed in the fatty acid profile. In general, meat analogs are perceived to be lower in saturated fatty acids, which are undesirable due to their association with diet-related diseases [13,31]. In the study by Harnack et al. [60], most of the plant-based ground meat analogs tested contained significantly lower levels of saturated fatty acids compared to ground beef. However, some products may contain similar or higher levels of saturated fatty acids, e.g., some plant-based burgers [25,30]. A key ingredient that results in higher saturated fatty acids in meat analogues is coconut fat and cocoa fat [10,30]. Meat analogues also have strengths: no cholesterol, lower energy value and high fibre content. It is, therefore, difficult to unequivocally confirm or deny the superiority of meat analogues in terms of nutritional value, as there is enormous variation in the composition of products in this food category. However, it is believed that meat analogues will be improved in the coming years in this respect as well [22,25]. 

Both producers and the scientific community focus primarily on the texture and flavour of meat analogues. However, there is little research that evaluates the safety of these products. Although plant-based ingredients are generally considered safer than meat, especially in terms of biohazards, there are some issues that are controversial [13]. The first aspect to consider is the impact of intensive processing on product quality. Meat analogues tend to contain large amounts of protein; thus, as in meat, there is a risk of the formation of toxic substances, such as heterocyclic aromatic amines (HAAs), N-nitrosamines or polycyclic aromatic hydrocarbons (PAHs) [9,17,22]. There is also a risk that valuable nutrients and health-promoting components in plant-based products may be lost during processing [6]. Factors that affect the safety of meat analogues also include pathogenic bacteria from raw materials, the presence of anti-nutritional components (e.g., protease inhibitors, phytic acid and oxalates), pesticide residues, heavy metal contamination and the allergenic potential of certain plant proteins [9,17]. 

It is also necessary to develop effective techniques for extending shelf life and ensuring the health safety of meat analogs. One method of preserving the quality of meat analogues is the use of antioxidants, especially of natural origin, mentioned earlier. However, the microbiological stability of the product is an equally important aspect affecting the safety of meat analogues. Various food additives are used to ensure this, but they are undesirable as consumers are increasingly looking for “clean label” products. For this reason, modern methods of preserving food products, especially meat, may become an interesting alternative to preservatives used so far. The effect of high temperatures creates the risk of unfavorable byproducts forming in meat analogs. For this reason, special attention should be paid to thermal methods using low temperature and non-thermal methods. Low-temperature methods that were applied in meat preservation are as follows: super-chilling, ultrarapid freezing, immersion vacuum cooling, hydrofluidization freezing, impingement freezing, electrostatic-assisted freezing and pressure-shift freezing. While non-thermal methods are acidic electrolyzed water coupled with high hydrostatic pressure and nonthermal plasma technique [61]. However, there is a need to verify the applicability of these methods for preservation of meat analogues. Attention should also be paid to consumer attitudes towards such product preservation techniques and the possible need for consumer education in this regard. Innovative packaging, e.g., containing active clay, may also be a promising tool to ensure shelf life and safety of meat analogues [61].

The success of meat analogues depends on consumers’ purchasing decisions; thus, changes may be necessary in such an important parameter as price. The basic raw material of meat analogues, such as plant protein, is significantly cheaper than meat. Nevertheless, the high costs of processing and other ingredients contribute to the high price of the final product. The inclusion of meat analogues in the diet, thus, poses an economic challenge for many consumers [22,29]. In order to encourage consumers to purchase meat analogues, it is also worth improving product marketing. Clarity and consistency of messages to consumers is an important issue, including the sustainability of production and the “naturalness” of the products offered [3,50].

**Figure 2:**
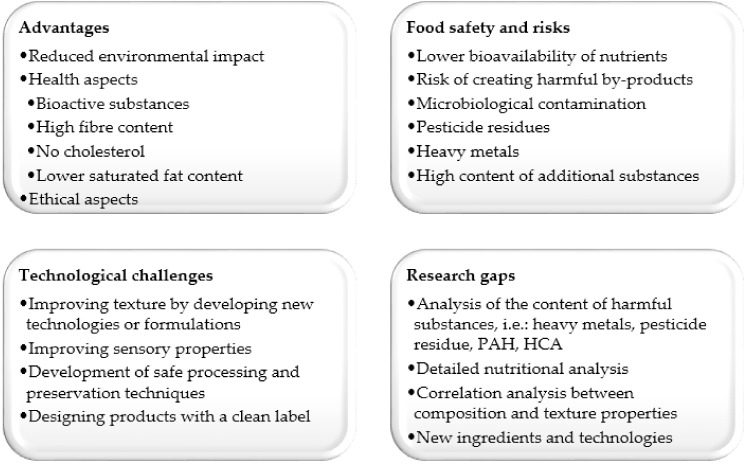
Advantages, risks, technological challenges, and research gaps associated with meat analogues (PAH- polycyclic aromatic hydrocarbons; HCA- heterocyclic aromatic amines).

The meat analogue sector has undergone dynamic development in recent times. Some researchers indicate that as a result of technological barriers, the development of meat analogues may be slower than expected [22]. However, there are still many issues to be regulated and improved. Future efforts by producers should focus not only on overcoming technological difficulties in terms of product texture and flavour but also on improving nutritional value, optimising the process to reduce costs and balancing environmental impacts. The safety aspect of analogue products should become a priority in both production and research. A summary of the strengths and weaknesses of meat analogues and the identified technology challenges and research gaps is presented in Figure 2.

## 6. Conclusions

Currently, meat analogues have mainly targeted people following a vegetarian or vegan diet. With increasing consumer awareness of the environmental impact of animal production, the unsustainability of the current food system and the health consequences of high meat consumption, the target group for analogue products has expanded to include flexitarians and meat eaters. The increased demand for meat alternatives on the market has contributed to the development of a number of texturing methods and formulations that have resulted in the latest generation of meat analogues possessing properties very similar to conventional meat products. Despite this, meat production and consumption are still at very high levels and are projected to increase further. This literature review has identified factors that act as barriers to increasing consumer consumption of meat analogues. A key aspect determining the low acceptance of analogue products is the unsatisfactory texture and sensory properties and the lack of confidence in the nutritional value and safety of these products. Thus, there is a great need to continue the search for innovative technological solutions and to focus attention on aspects of concern to consumers such as the following: sodium content and lack of “clean label”. In the research area, there is a shortage of comprehensive research on meat analogues covering, apart from their technological properties, also their safety related to, inter alia: possible contamination with heavy metals and toxic substances, microbiological stability, residues of plant protection products and content of anti-nutritional components.

## Figures and Tables

**Table 1:** Composition of meat analogues in scientific research.

Product Type	Composition	Tested Parameters	Reference
meat analogue	soy protein concentrate, wheat gluten, vegetable oil, pumpkin powder, wheat starch, salt	protein content, moisture, pH, colour, textural properties, sensory properties, microscopy, protein solubility	Chiang et al. 2019 [36]
chicken analogue	wheat gluten, soy protein isolate, water, soybean oil, wheat starch	protein content, moisture, pH, textural properties analysis, scanning electron microscopy, total amino acids, protein solubility	Chiang et al. 2021 [37]
meat analogue	soy protein isolate, wheat gluten, water, salt	pH, maximum swelling, water holding capacity, ionic strength	Cornet et al. 2021 [33]
meat analogue	pea protein isolates, defatted soy protein isolates, oat protein	protein content, texture profile analysis, water absorption, oil absorption, sensory analysis	De Angelis et al. 2020 [11]
meat analogue	protein concentrate from faba beans	specific mechanical energy (SME), differential scanning calorimetry (DSC), colour, cooking yield, water binding capacity, oil binding capacity, textural properties, sensory evaluation	do Carmo et al. 2021 [38]
meat analogue	soy protein isolate, wheat gluten, and natural flavor powder	volatile compounds profile, scanning electron microscopy, low-field nuclear magnetic resonance (NMR), fourier transform-infrared (FT-IR) spectroscopy	Guo et al. 2020 [14]
meat analogue	rapeseed protein concentrate, soy protein concentrate, wheat gluten, water, salt	macrostructure, color, tensile strength, scanning electron microscopy, confocal laser scanning microscopy, X-ray microtomography	Jia et al. 2021 [39]
high moisture extruted (HME) protein	soy protein isolate, whey protein concentrate	texture, cryo-imaging, micro-CT, rheological measurements, scanning electron microscopy	Wittek et al. 2021 [40]
